# Supplementary material for: Histological E-data Registration in rodent Brain Spaces
Source: eLife. 2023 Jan 13;12:e83496. doi: 10.7554/eLife.83496 (PMC9904758; doi:10.7554/eLife.83496)
Supplement: Supplementary file 1. — The name and year of publication for each tool kit are shown at top, and the functionalities of each tool kit are listed below. [file elife-83496-supp1.docx]

| **Supplementary Table 1** | | | | | |
| --- | --- | --- | --- | --- | --- |
| Functions/software | HERBS | TRACER | Brainrender | Brainreg | SHARPTRACK |
| Year | 2022 | 2021 | 2021 | 2020 | 2018 |
| Python | ✓ | ✓ | ✓ | ✓ |  |
| GUI | ✓ |  | ✓ | ✓ | ✓ |
| Histology data | ✓ | ✓ |  |  | ✓ |
| Whole-Brain data | △^1^ |  | ✓ | ✓ | ✓ |
| Pre-surgical registration | ✓ | ✓ |  |  | ✓ |
| Post-surgical registration | ✓ | ✓ |  | ✓ | ✓ |
| 3D Visualization tool | ✓ | ✓ | ✓ | ✓ | ✓ |
| In-built atlases | ✓ | ✓ | ▽^2^ | ▽ | ✓ |

^1^ In developement

^2^ Plugin required

**Supplementary Table 1.**Table summarizing the currently available anatomical tool kits for use with rodents. The name and year of publication for each tool kit are shown at top, and the functionalities of each tool kit are listed below.
